# Struvite Precipitation as a Means of Recovering Nutrients and Mitigating Ammonia Toxicity in a Two-Stage Anaerobic Digester Treating Protein-Rich Feedstocks

**DOI:** 10.3390/molecules21081011

**Published:** 2016-08-03

**Authors:** Shunli Wang, Gary L. Hawkins, Brian H. Kiepper, Keshav C. Das

**Affiliations:** 1 College of Engineering, University of Georgia, Athens, GA 30602, USA; wshl2000@uga.edu; 2 College of Agricultural and Environmental Sciences, University of Georgia, Athens, GA 30602, USA; ghawkins@uga.edu (G.L.H.); bkiepper@uga.edu (B.H.K.)

**Keywords:** struvite, anaerobic digestion, ammonia, precipitation, protein-rich wastes

## Abstract

Accumulation of ammonia, measured as total ammonia nitrogen (TAN), a product of protein decomposition in slaughterhouse wastes, inhibits the anaerobic digestion process, reducing digester productivity and leading to failure. Struvite precipitation (SP) is an effective means to remove TAN and enhance the buffering of substrates. Different Mg and P sources were evaluated as reactants in SP in acidogenic digester effluents to reduce its TAN levels. In order to measure impact of TAN removal, a standard biochemical methane potential (BMP) test was conducted to measure methane yield from treatments that had the highest TAN reductions. SP results showed 6 of 9 reagent combinations resulted in greater than 70% TAN removal. The BMP results indicated that SP treatment by adding Mg(OH)_2_ and H_3_PO_4_ resulted in 57.6% nitrogen recovery and 41.7% increase in methane yield relative to the substrate without SP. SP is an effective technology to improve nutrient recovery and methane production from the anaerobic digestion of protein-rich feedstocks.

## 1. Introduction

Anaerobic digestion (AD) is an attractive technology to treat high-strength slaughterhouse wastes as it provides energy recovery (methane), nutrient recovery (nitrogen and phosphorus), and pathogen destruction [1,2]. Energy recovered in the form of methane by treating slaughterhouse wastes in AD was reported to be 1300 Megajoule (MJ) per bovine, 140 MJ per pig and 1.3 MJ per broiler [3,4,5]. As much as 23.0 g of nutrients (total of nitrogen, phosphorus and potassium) per bird are potentially recoverable from poultry slaughterhouse wastes [5]. With respect to pathogen destruction, both fecal coliform and salmonella were reportedly completely eradicated in a thermophilic digester (50 °C), while 99.9% and 90%–99% of oocysts of *Eimeria tenella* were inactivated in a thermophilic digester and a mesophilic digester, respectively [2].

In AD, proteins and lipids are hydrolyzed and acidified to intermediates including hydrogen, NH_3_ (measured as total ammonia nitrogen or TAN) and volatile fatty acids (VFA) in the acidogenic stage of the process. Hydrogen and VFAs are then converted to methane and carbon dioxide by a different group of bacteria in the methanogenic stage. High concentrations of TAN are known to severely inhibit methanogenic microorganisms, reducing digester performance and leading to failure. Strategies of TAN management are needed when treating high protein wastes in AD.

One of the best management strategies is the use of a two-stage AD system where acidogenesis (and the production of TAN and VFA) are physically separated from methanogenesis to reduce TAN and VFA inhibition of methanogens. However, high TAN concentrations (over 2.0 g·L^−1^) produced during acidogenesis of slaughterhouse waste proteins [6] are well above the known inhibitory level of 1.5 g·L^−1^ [7]. Reduction of TAN in the substrate through external intervention can therefore enhance digester performance. Considering TAN is not biologically removed in AD, removing it via physical stripping or chemical precipitation from the acidogenic digester effluent could be a way to reduce concentrations and associated inhibition downstream in the methanogenic digester.

Magnesium ammonium phosphate (MAP) commonly known as struvite is a compound with low aqueous solubility under alkaline conditions [8]. When concentrations of TAN, magnesium and phosphorus reached critical values, struvite is naturally formed and is known to attach to and clog pipes in wastewater treatment. Struvite is also a slow release fertilizer with commercial value and its precipitation from different high-TAN wastewaters, including AD effluents, has been studied extensively [9,10]. Çelen and Türker [9] reported 50% to 90% TAN removal from AD effluents through struvite precipitation (SP) using H_3_PO_4_ and MgO or MgCl_2_ under short reaction times (~10 min), high pH (~9.0) and room temperature (25 °C). In order to improve AD performance by TAN reduction, in some cases magnesium (Mg) and phosphorus (P) were directly added to the digester [8,11]. Lee et al. [8] reported reduction of 67% TAN and 73% of P in the substrate after adding MgCl_2_ to a single-stage food waste digester thus increasing methane yields from 180 to 290 mL·g^−1^ COD_added_. Romero-Güiza et al. [11] added a low-grade MgO byproduct to a single-stage digester treating pig manure and found methane yields increased from 130 mL·g^−1^ VS_added_ (before MgO addition) to 190 mL·g^−1^ VS_added_ (after 30 kg MgO·m^−3^ addition). SP directly in the digester has been shown to significantly improve methane yield, however, accumulation and deposit of struvite within the digester gradually reduce the effective volume of the digester. In addition, once settled inside digesters the precipitate is difficult to remove. Establishing a struvite precipitator external to the digester, between the 1st and 2nd stages of a two-stage digester, is a practical way to remove TAN from substrates before feeding the substrate to the 2nd stage methanogenic digester.

Integrating an external SP with a two-stage digester treating high-protein substrates can result in nitrogen (N) recovery, reduced TAN inhibition, and increased buffering in the methanogenic digester. In order to evaluate such a process and its effectiveness a study was conducted with the following goals: (1) identify the most suitable reagents that maximize N recovery while minimizing residual methanogenic toxins; (2) test Mg and P sources and quantify TAN removal in the effluent of an acidogenic digester; and (3) perform a biochemical methane potential (BMP) test to quantify methane yields from the treated substrates, compared to controls that did not receive SP.

## 2. Results and Discussions

### 2.1. Selection of Mg and P Sources for SP Test

The Mg and P sources that can be used in SP and residual toxins that could remain, along with their inhibition thresholds in AD, are summarized in Table 1. Since the substrates after SP are fed to the methanogenic digester, the criteria for selecting Mg and P sources included both highest TAN removal and least amount of residual toxins (e.g., ions and elements such as N, S, Na^+^, K^+^ or Cl^−^) that could potentially inhibit methanogenic activity. Since these elements are highly soluble in the substrates and will remain in the treated substrates, the reagent dosages would have to be calculated to minimize inhibition of methanogenesis. Therefore, based on the minimal toxic residue criteria, MgO, Mg(OH)_2_, MgCO_3_, and MgHPO_4_ were selected as Mg sources, and H_3_PO_4_ and NaH_2_PO_4_ were selected as P sources for further evaluation as reagents in SP of acidogenic effluents.

### 2.2. Experiment 1—Optimization of Struvite Precipitation

TAN of substrates was found to be 1.3 ± 0.1 g·L^−1^, a value approaching inhibition levels for methanogenesis, potentially leading to lower process efficiency and TAN removal is expected to reduce these impacts (Table 2). Concentrations of Ca, P and Mg in the substrate were too low to contribute to SP in any significant way as found in a preliminary test (Table S1).

Treatments G1 and G5 using H_3_PO_4_ had TAN removal of more than 70% (Table 3), while G2 and G6 using NaH_2_PO_4_ were less than 60%. As H_3_PO_4_ is a relatively strong acid, MgO and Mg(OH)_2_ are more readily dissolved in it providing more active Mg^2+^ for SP relative to their mixtures with NaH_2_PO_4_ [17,18]. TAN removals in G1 and G5 treatments were higher than those reported in the literature treating different substrates under similar conditions. In particular, TAN removal in G1 was 77.5%, compared to 54.4% and 25.7% removal reported by Celen and Türker [9] and Yetilmezsoy and Sapci-Zengin [19], respectively. Such large differences could be caused by higher availability of Mg^2+^ formed from the dissolution of MgO that is enhanced by the lower pH in this study (pH = 6.4) relative to that in the literature where pH were 7.9 [9] and 7.95 [19]. Substrates used in the above studies had a higher pH of 7.9 and after adding equimolar H_3_PO_4_, MgO dissolved in the substrate was relatively small. TAN removals using Mg(OH)_2_ were comparable to those using MgO when combining different P sources. Treatments G2 and G6 using NaH_2_PO_4_ had lower TAN removal of 50.5% to 58.8%. These results indicate higher removal of TAN compared to Li et al. [20] who treated pharmaceutical wastewater with pH of 12.2 and TAN of 1.12 g·L^−1^ by adding MgO and NaH_2_PO_4_ (Mg^2+^:NH_4_-N:PO_4_-P = 1:1:1) and maintaining pH of 9.0 for 15 min under well mixed conditions. The TAN removal in the above study was reported to be less than 40%, which was lower than the 50.5% TAN removal observed in this study. Based on the work of Celen and Turker [9], it is known that TAN removal via SP is complete within 5 min when operating in a pH between 7.5 and 9.0. Therefore, the 20-min reaction time used in this study relative to the 15-min used by Li et al [20] was not the reason for difference in performance. The higher TAN removal obtained, is a result of the lower pH in the used substrate (pH = 6.4) relative to pH in Li et al. (pH = 12.2). Treatment G6 had similar TAN removal to G2, because of the similar chemical reaction of MgO and Mg(OH)_2_ in SP.

Treatments of G3 and G4 containing MgCl_2_ had higher TAN removals of 88.3%–89.0%. These values are in the same range as reported in other studies treating different wastewaters using SP [9,21,22]. MgCl_2_ is a highly water soluble compound and provides the highest ion concentrations of Mg^2+^ without the addition of acids, resulting in high TAN removal regardless of the strength of acids used (e.g., H_3_PO_4_).

Treatments containing MgCO_3_ also had similar high TAN removals in the range of 75.9% to 76.5%. In contrast to treatments using less soluble MgO and Mg(OH)_2_, with MgCO_3_, large amounts of CO_2_ bubbles were observed when reagents were added to the substrates. This bubbling of CO_2_ resulted in some mixing of the solution and potentially enhanced mass transfer and ionization of MgCO_3_ to Mg^2+^.

It was surprising that treatment G9 containing MgHPO_4_ resulted in very low TAN removal (5.2%). This result is not in agreement with previously reported results in the literature. For example, Sugiyama et al. [23] reported TAN removals of 49% and 77% for 1 and 3 h of SP reaction time, respectively, using MgHPO_4_ at pH of 8 and temperature of 25 °C using an equimolar ratio of NH_4_Cl to MgHPO_4_. This difference in performance could be explained by the fact that the MgHPO_4_ powder was dissolved in the HCl solution first and then added to the substrate. The Mg^2+^ and PO_4_^3−^ ions were released in the acid solution and possibly precipitated in the form of compounds such as Mg_3_(PO_4_)_2_ instead of struvite [24]. A dark colored precipitate was seen with treatment G9, different from other treatments, which had a white precipitate characteristic of struvite, showing possibility of other compounds having precipitated in addition to struvite. A longer reaction time is probably necessary to form struvite when using MgHPO_4_ as the sole Mg and P reagent. These possibilities need further validation.

TAN was also potentially removed by free ammonia volatilization from the substrate, which was continuously stirred at high pH and temperatures [7,19]. In this study, which was conducted at 25 °C, 20 min, and pH 8.5, only 15.2% of TAN was in the form of free ammonia, calculated from the equation in Cuetos et al. [25]. Therefore, considering the high solubility of ammonia in water, ammonia volatilization is not expected to have a significant impact on TAN reduction relative to TAN removal by SP [19,20].

### 2.3. Experiment 2—Biochemical Methane Potential of TAN Reduced Substrates

In order to generate sufficient substrate for BMP tests, larger volumes of substrates were treated with SP reagents following the procedure of experiment 1. Characteristics of substrates to be used for BMP testing (before and after SP) are shown in Table 4.

Untreated substrates had very low concentrations of Mg, Na and P to impact SP in any appreciable manner. There was also an absence of micronutrients at concentrations toxic to AD [15,26]. After SP, no significant COD change was observed between treated and untreated substrates. Previous researchers have reported COD reduction in wastewaters after SP ranging between 10% and 20% [20] and 22.4% to 53.3% [19]. These COD reductions are typically caused by co-precipitation of organics and struvite [19]. In this study, because of prior treatment through acidogenesis, organics were mostly converted to soluble VFA and did not participate in the co-precipitation. TAN removal of each treated substrate ranged between 57.4% and 93.8%. In treatment G1 (MgO treatment), it was observed TAN removal of 57.4%, which was lower than the 77.5% removal observed in experiment 1. For other treatments, results of TAN removals were comparable between experiments 1 and 2. The SP treatment substantially increased TS and VS of substrate because of salt residues such as Mg(OH)_2_, and NaH_2_PO_4_ [27,28], however, it did not appreciably change feedstock rheology which allowed for the treated substrate to be easily pumped into the liquid-state anaerobic digester. The C/N of substrates increased from 5.0 to 22.7 as a result of TAN removal, providing the additional benefit of nutrient balance in AD. It is not surprising that residues of certain reagent elements such as Na, P and Mg remained in the substrates. After SP, Na concentrations were 3526 to 7432 mg·L^−1^ depending on the amount of NaOH added for pH adjustment. High Na concentrations in substrates can potentially inhibit AD [15]. However, inhibition can be alleviated if microorganisms are properly acclimatized to the conditions [29]. Residual substrates had equimolar ratios of TAN and P, but less than equimolar contents of Mg. This suggests that TAN and P removal were directly in the form of struvite, however, in addition to contributing to struvite, Mg was also removed through the precipitation in the form of other compounds. Magnesium was reported as the limiting factor in SP in previous studies (e.g., [30]), confirming the observations in this study. Residual Mg in the treated substrates ranged from 23.96 to 90.44 mg·L^−1^, far less than the moderately inhibitory threshold of 1000–1500 mg·L^−1^ [31]. Residual total P concentrations were in the range of 195 to 1246 mg·L^−1^. P is an important requirement for living microorganisms and plays a vital role in their growth and metabolism [32]. Based on the authors’ best knowledge, few studies have looked at the impact of high P concentrations on AD. Wang et al. [32] and Lei et al. [33] reported preferred concentrations of 414 and 465 mg P·L^−1^, respectively, in the anaerobic digestion of different substrates. The residual P in the substrates in this experiments (with the exception of G3) was relatively high and can be reduced by adding excess Mg in SP. Due to different final TAN concentrations in different treatments that may itself cause an impact, the TAN concentration was normalized to 0.6 g·L^−1^ in all treatments prior to conducting the BMP test (Table 5).

**Figure 1:**
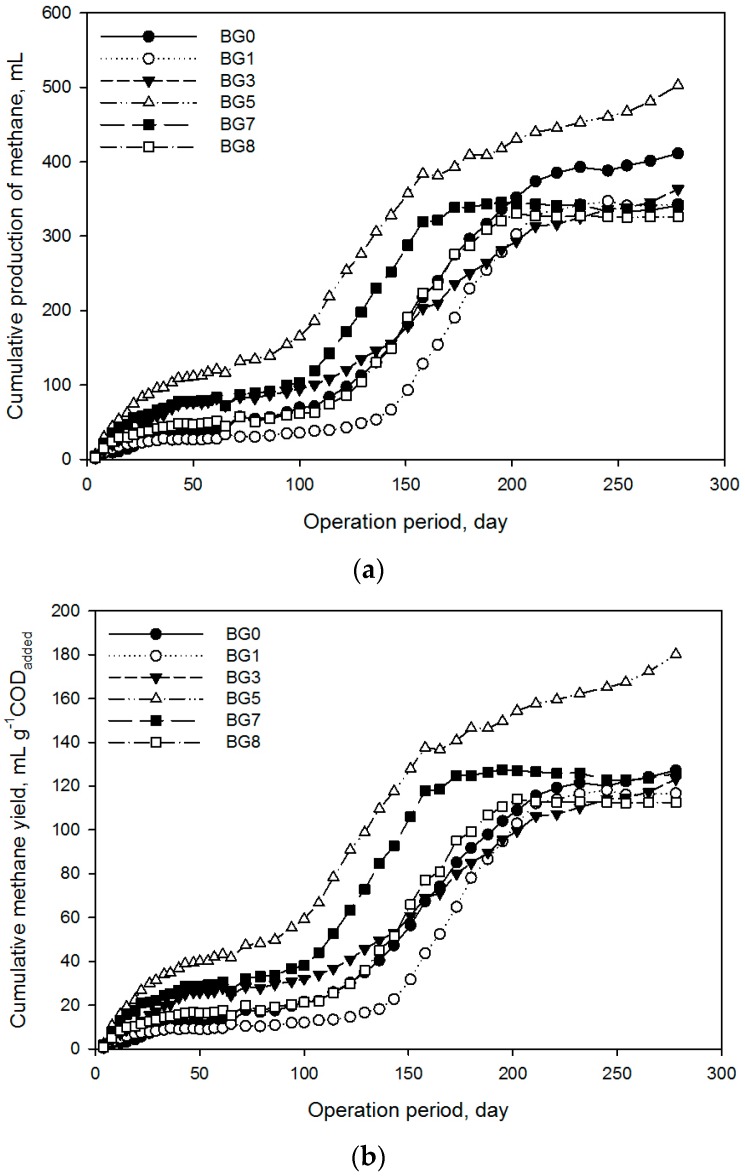
Cumulative methane production (**a**) and yield (**b**) of struvite precipitated substrates from the acidogenic digester treating poultry blood and wastewaters. Each point denotes the average value of three replicates.

**Figure 2:**
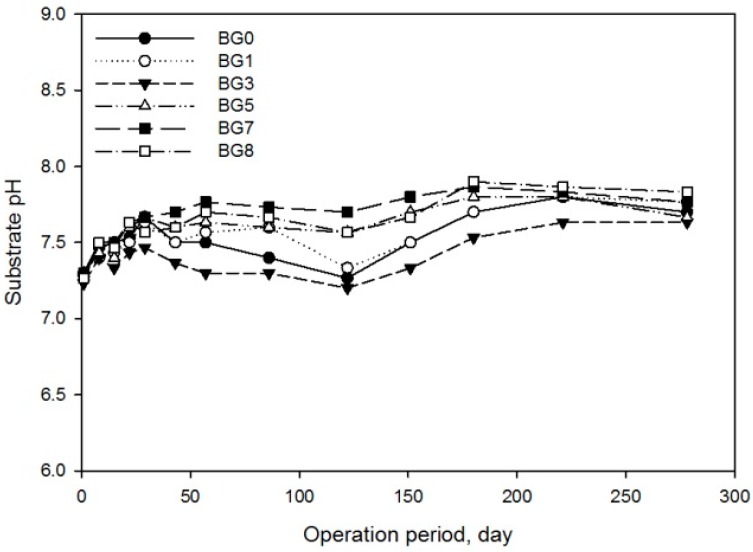
pH changes in the BMP treatments. Each point denotes the average of three replicates.

Cumulative methane productions and yields are presented in Figure 1. As expected, the control with only inoculum had negligible methane production (3 mL, Figure S1) compared to other treatments, confirming the very low biodegradable organic fraction in the inoculum. All treatments showed long lag phases in the range of 30 to 140 days, which is longer than reported in other similar work [1,5]. The inoculum from the methanogenic digester operated at 26 °C may also have required some time to adapt to the higher test temperature of 38 °C. The pH in all treatments increased from 7.3 to around 7.6 and then stabilized for the rest of the BMP test, with a slight pH drop that occurred at the 122nd day (Figure 2). The buffering effects to maintain an acceptable pH were from TAN and P compounds including solution of Na_3_PO_4_, Na_2_HPO_4_ or NaH_2_PO_4_. It is known that these compounds have a high buffering capacity and are often used to make common buffering reagents.

Table 6 shows performance parameters of each treatment. The BMP results of treated substrate BG5 showed improvement over the untreated control, while other treatments had no significant difference. Treatment BG0 that contained untreated substrate had a relatively longer lag phase than most other treatments. However, methane yield from BG0 was in the range of most other treatments with the exception of BG5. These results indicate that SP shortened the adaptation time. Among BMP of treated substrates, COD and TAN of feedstocks had negligible differences (Table 7). The major differences impacting the BMP test were residual Cl^−^, Na (in form of sodium ion) and P (total phosphorus). Chloride toxicity was reported at a concentration of 5500 mg·L^−1^ in the case of 0.6 g·L^−1^ tannin input in AD [16]. Since measured levels in the treatments were lower (335 to 3026 mg·L^−1^), it was concluded that chloride toxicity may not have been a factor in this experiment. Inhibition level of Na is known to be 3500 mg·L^−1^ in AD [31] and since measured Na levels in all treatments were lower, no negative impacts were anticipated.

Treatment BG5 had the shortest lag phase (8.7 days) and highest methane yield (180.2 mL·g^−1^ COD_added_) in this experiment. BG1 with 897 mg·L^−1^ of P had the longest lag phase of 125.7 days, suggesting that microorganisms required a long adaption period to adjust to high P substrate. The best performance of BG5 showed that 623 mg·L^−1^ of P was an optimum level, compared to the 408 to 422 mg·L^−1^ of P in treatments of BG7 and BG8. This result is slightly higher than results in a previous study [33] that reported the optimum P concentration was 465 mg·L^−1^. COD removal of all treatments ranged from 42% to 65%.

### 2.4. Optimizatiom of SP Treatment

In SP treatments, TAN was significantly removed from substrates, which improved anaerobic process by shortening the lag phase time or increasing methane yields. However, residuals such as Na, Cl^−^, Mg (total magnesium) and P were introduced at different levels to the substrate. If not properly managed, the high concentrations of such elements can result in inhibition of AD, economic loss of P as a valuable nutrient, and potential eutrophication of water bodies if the P was discharged to the environment.

Actions should be taken to minimize the residual Na and P in the treated substrates lower than the inhibition levels. Adding extra Mg and reducing the SP reaction pH can facilitate P precipitation and reduce usage of NaOH for pH adjustment, respectively. Because of the low solubility of most Mg sources and high solubility of P sources, the addition of excess Mg source is required to provide more active Mg^2+^ to improve SP. Celen and Türker [9] reported that when TAN and P are present in equimolar concentrations, SP was improved when Mg concentration was increased. Yetilmezsoy and Sapci-Zengin [19] also concluded that TAN removal efficiency increased when excess Mg was added using MgCl_2_. Few studies have looked at using MgCO_3_ in SP in wastewaters, probably because of its higher price compared to MgO, MgCl_2_ or Mg(OH)_2_. The Mg provided by MgO is cheaper than that of MgCl_2_ [9]. To keep cost down, low-grade MgO can be used to remove the TAN (e.g., [11]). The residual Na from NaOH addition can be decreased if the reaction pH is controlled at 7.5 or 8.0 without seriously impact of SP effect [9].

Treatment BG5 had 41.7% more methane yield than treatment BG0 and obtained 57.6% nitrogen recovery in the form of struvite (calculated from Table 4 and Table 5). SP in other treatments recovered more than 57% nitrogen but did not seem to have much impact on methane yields, though the longest lag phase occurred in treatment BG1. Finally, based on nitrogen recovery performance and BMP it is concluded that BG5 was the best candidate among those tested and is recommended.

### 2.5. Economic Analysis

**Figure 3:**
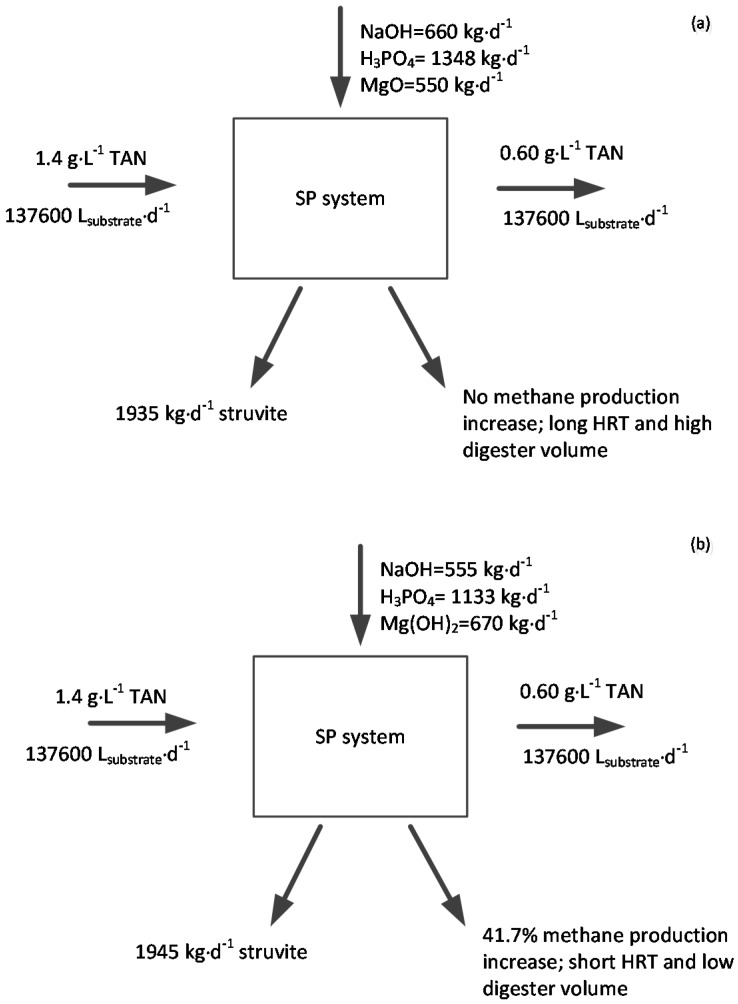
Simplified diagram for the calculation of economic benefits of SP using MgO (**a**) and Mg(OH)_2_ (**b**) as Mg source.

Economic benefits were estimated to evaluate feasibility of SP in AD of poultry slaughterhouse blood wastes. Figure 3 and Table 8 show a chemical mass flow and the estimated economic benefits from SP for downstream methanogenesis, respectively. The net economic benefits from struvite and additional methane sale in the SP system using MgO and Mg(OH)_2_ are $434.42 and $262.13–738.10 d^−1^, respectively. The cost of Mg accounts for 11.2%–39.6% of output benefit, however, additional methane sale only accounts for 0–2.2%. Hence, using low-cost Mg sources could significantly increase economic benefits of this process. Several researchers [11,34] suggest use of cheaper MgO/Mg(OH)_2_ sources such as low-grade MgO and Mg(OH)_2_ slurry to improve economic benefits and enhance process feasibility in large scale AD. The feasibility of using low-cost Mg can be evaluated using methods introduced in this study or by software simulation [35] to avoid residual toxins in the treated substrates that could inhibit downstream methanogenesis. In addition, it should be mentioned that the system using Mg(OH)_2_ showed a shorter lag phase in the BMP test (Table 6), suggesting shorter HRT in continuous operation. This will allow for a smaller digester, thus reducing capital costs. Comprehensive evaluations, including the price of Mg, toxicity in methanogenesis and methanogenic digester capital costs, should be made before choosing the Mg source in this system.

## 3. Materials and Methods

### 3.1. Substrates

Acidogenic digester effluents were collected from three identical 40-L pilot acidogenic digesters treating co-substrates of poultry slaughterhouse wastewaters (PPWW) and poultry blood (3:1, *v*/*v*) that was diluted 50% with de-chlorinated water to reduce its initial organic strength. Each digester was stirred by recirculating substrates from the bottom to the top using a diaphragm pump, at an approximate flow of 5.7 L·min^−1^, that was operated by a programmable timer for 15-min on and 15-min off. The digesters had been actively operating at mesophilic conditions (26 ± 2 °C) for approximately six months at the point of sampling. Collected samples were stored in a refrigerator (≤4 °C) for further testing prior to use.

### 3.2. Experiment 1-Optimization of Struvite Precipitation

#### 3.2.1. Reagents

Five Mg compounds, namely, MgO, MgCl_2_·6H_2_O, Mg(OH)_2_, MgCO_3_ and MgHPO_4_·3H_2_O and three P compounds, namely, 85% H_3_PO_4_, NaH_2_PO_4_·H_2_O and MgHPO_4_·3H_2_O were used in this experiment.

These compounds were selected based on ease of market availability and the low concentration of residual toxins that remain after reactions (Table 9). A solution of 300 g NaOH·L^−1^ was prepared and used for pH adjustment in the SP protocol. Nine combinations of reagents, namely, MgO + 85% H_3_PO_4_ (G1), MgO + NaH_2_PO_4_·H_2_O (G2), MgCl_2_·6H_2_O + 85% H_3_PO_4_ (G3), MgCl_2_·6H_2_O + NaH_2_PO_4_·H_2_O (G4), Mg(OH)_2_ + 85% H_3_PO_4_ (G5), Mg(OH)_2_ + NaH_2_PO_4_·H_2_O (G6), MgCO_3_ + 85% H_3_PO_4_ (G7), MgCO_3_ + NaH_2_PO_4_·H_2_O (G8), and MgHPO_4_·3H_2_O (G9), were evaluated to rank their performance in TAN removal.

#### 3.2.2. Struvite Precipitation Protocol

Amount of reagents required for 500 mL of substrate was calculated so as to result in a molar ratio of 1:1:1 of Mg:NH_4_:PO_4_. The substrate TAN was measured to be 1.36 g·L^−1^, while Mg nor P in the feedstock were measured because expected amounts in the substrate were known to be relatively low (based on values in the range of 0.006 to 0.0655 g·L^−1^ measured during preliminary studies). Reagents and substrates were reacted in 500-mL Erlenmeyer flasks with aluminum covers and a magnetic stir bar placed in the flask to stir the substrates at 500 rpm on a stir plate. A volume equal to 510 mL substrates was placed in the Erlenmeyer flask and heated to room temperature in 1 to 3 min using a water bath at 50 °C. A 10-mL aliquot of substrates was sampled at the start of the experiment (T0). Subsequently, Mg and P reagents were added to the substrates in sequence (MgHPO_4_ was dissolved using small amount of diluted HCl solution and then added to the substrate). After adjusting substrate pH to 8.5, 10-mL substrate was sampled after the 20 min (T20) time point. A 1-mL aliquot from the T0 samples and from the supernatants of the T20 samples (1 h after sample collection) was analyzed for TAN concentrations. The SP after 20 min of reaction time was considered complete based on previous studies [9,19]. Each treatment was replicated twice.

### 3.3. Experiment 2- Biochemical Methane Potential of TAN Reduced Substrates

#### 3.3.1. Treatments Used in Experiments

Due to high levels of TAN removal (>70%) observed with different reagent combinations, treatments G1, G5, G7 and G8 were selected for further BMP analyses. Treatment G3 was also selected because of highest TAN removal and the popular use of these reagents in previously reported SP studies. A volume equal to 1700 mL of substrates was placed in a 2000-mL Erlenmeyer flask and processed exactly as was done in the SP experiment described earlier. After 20 min, stirring was stopped and the substrate was allowed to stand in the flask for one hour to allow for gravitational settling of struvite precipitates. The supernatant was poured slowly into storage bottles and stored in a refrigerator (4 °C) for further use in chemical analyses and BMP testing.

#### 3.3.2. Inoculum

Inoculum used in the BMP tests was collected from a 87-L methanogenic digester treating pH adjusted effluent (pH 7.2–7.4) from a 40-L acidogenic digester. This methanogenic digester was operated at an OLR of 0.4 g COD·L^−1^·d^−1^ (corresponding to HRT of 37.8 days) and had been actively operating at mesophilic conditions (26 ± 2 °C) for approximately four months when inoculum was collected. The inoculum was placed in a pre-incubated anaerobic digester at 38 °C for three days to deplete any un-degraded biological residues present in the inoculum before use in the BMP assay, as suggested by Angelidaki et al. [41]. The characteristics of the inoculum were 2.9 ± 0.1 g·L^−1^ of TS, 1.3 ± 0.1 g·L^−1^ of VS, 1.4 ± 0.0 g·L^−1^ of TAN, and 1.6 ± 0.4 g·L^−1^ of COD.

#### 3.3.3. Biochemical Methane Potential (BMP) Assay

The BMP was tested using 500-mL batch anaerobic digesters with an effective substrate volume of 300 mL incubated at 38 °C. Substrate to inoculum ratio was 70/30 (*v*/*v*) which corresponded to 3 g-VS untreated substrate to 1 g-VS inoculum [42]. Because different treatments had different final TAN concentrations after SP, before use in the BMP test, they were mixed with untreated substrate (which had higher TAN concentration) to normalize all treatment TAN concentrations to 0.6 g·L^−1^. The pH of the mixture of untreated/treated substrates and inoculum was then adjusted to 7.3 using HCl solution before placing in the BMP test digesters. The headspace of the digesters were purged using N_2_ gas and sealed using butyl rubber stoppers and aluminum crimps. To obtain a complete profile, the BMP digesters were incubated for 278 days with treatment and controls replicated three times. Blank digesters of triplicate were run using the inoculum and DI water replacing substrates. Methane yields were calculated at normal temperature and pressure (NTP) and expressed as mL CH_4_·g^−1^ COD_added_. Biogas production and methane concentrations in the biogas were measured every 4 to 12 days depending on the level of activity. The pH was measured every 7 to 57 days by collecting 1-mL sample from serum bottles and measuring pH using a laboratory pH probe. Small amounts of substrates (12 mL) were taken from each digester for pH measurement in the whole experiment period. The pH was measured more frequently, every 7 days in the first month, and less frequently close to the end of experiment, 57 days.

#### 3.3.4. Calculation of Cumulative Methane Production

The cumulative methane production was calculated using the following equation

(1) $$M_{n}=\sum_{1}^{n}(m_{n}\times C_{n})+H\times C_{n}$$

where *M_n_* is the cumulative methane production till the *n*th day (mL); *m_n_* is the biogas production on the *n*th day (mL); *C_n_* is the methane content in biogas on the *n*th day; *H* is the headspace in the bottle and measured as 239 mL.

In this equation, $\sum_{1}^{n}\;(m_{n}\times C_{n})$ is the cumulative methane volume measured by discharging biogas and testing methane percentage in biogas on the *n*th day and $H\times C_{n}$ is the methane volume in the bottle headspace on the *n*th day.

#### 3.3.5. Modeling the Kinetics of Methane Production

A modified Gompertz model was used to model cumulative methane production during the incubation period [5] as shown below:

(2) $$M=P_{m}\times \exp\left\{-\exp\left[\frac{R_{m}\times e}{P_{m}}(\lambda -t)+1\right]\right\}$$

where *M* is the cumulative methane production (mL); *e* is 2.718282; *R_m_* is the maximum specific methane production rate (mL·d^−1^); *P_m_* is methane production potential (mL); and λ is the lag phase time (days).

#### 3.3.6. Analytical Methods

The pH was measured using an Accumet portable AP61 pH meter (Fisher Scientific, Hampton, NH, USA). COD was measured in samples that were diluted 100-fold using the HACH method 8000 (HACH, Loveland, CO, USA). Total solid (TS), volatile solid (VS) and total suspended solid (TSS) were analyzed following standard laboratory methods used in the wastewater industry [43]. TS and VS were measured by drying 30-mL samples at 105 °C for 24 h and then burning at 550 °C for 1 h. TSS was measured by filtering 10 mL samples through a 1.6-μm filter and drying the filter with residue at 105 °C for 24 h. Total Nitrogen (TN) and TAN were measured on a 100-fold diluted sample using HACH method 10072 and 10031, respectively (HACH, Loveland, CO, USA). Micronutrients and chloride concentration of samples were analyzed at the Soil, Plant, and Water Analysis Laboratory of University of Georgia. For micronutrients analysis, 0.5-g or 1-mL sample was added to 5 mL of concentrated HNO_3_ and digested in a microwave oven following the USEPA method 3051A. The digested solutions were analyzed using an Inductively Coupled Plasma-Optical Emission Spectrometer (ICP-OES) (Spectro Arcos FHS16 AMETEK ICP-OES, SPECTRO Analytical Instruments Inc., Mahwah, NJ, USA). For Cl^−^ analysis, the samples were diluted 100 to 2000 fold using DI water and filtered through a 0.45-μm syringe filter. Further analyses were carried out in an ion chromatograph (Metrohm 861 Advanced Compact IC, Metrohm Ltd., Herisau, Switzerland) running at a flow rate of 0.7 mL·min^−1^. The CHNS concentrations were measured using a FLASH 2000 CHNS-O analyzer (Thermo Fisher Scientific, Waltham, MA, USA) on freeze dried samples. Approximately 1 mg dried samples were weighed in tin capsules and placed in the instrument that quantified elements by combustion and detection of elements in the off gases. Volume of biogas produced was measured by volume-displacement in a Eudiometer water column (Selutec, Hechingen, Germany), while methane concentration was measured using a GC-FID (SRI310C, SRI Instruments, Torrance, CA, USA). The method used a stainless steel column (80/100 HayeSep D 6’ × 1/8’’, Supelco, Bellefonte, PA, USA); oven and detector temperatures of 40 °C and 380 °C, respectively; Carrier gas, fuel gas and oxidizing gas were helium (10 mL·min^−1^), hydrogen (25 mL·min^−1^) and air (250 mL·min^−1^), respectively. Total biogas volume generated was measured by puncturing the rubber lid of each digester with a needle and syringe, which was connected to a Eudiometer by airtight tubing. Biogas samples (0.1-mL) were taken from the headspace of each digester using a gastight syringe and tested in the GC.

#### 3.3.7. Statistical and Regression Method

A one-way ANOVA and Tukey HSD test (JMP software, Pro 10, SAS Institute, Cary, NC, USA) were used to compare methane yields and COD and TAN removal from different substrates in the BMP study. Differences between treatments were considered significant at *p* ≤ 0.05. The nonlinear regression of the modified Gompertz model was performed using SigmaPlot 12.

#### 3.3.8. Economic Analysis

A typical poultry processing plant processes 200,000 birds daily and discharges 17,200 kg blood when 50% of the total blood generated is collected [44]. Blood can be diluted using poultry slaughterhouse wastewaters and fresh water at a volumetric ratio of 1:3:4 (blood: wastewaters: fresh water) and fed into digesters as described in the experiment. Assuming the diluted blood has the same density as water, 137,600 L·d^−1^ acidogenic digester effluent containing 1.4 g·L^−1^ TAN enter the proposed SP system. In the system using MgO as Mg source, all the effluent is treated (BG1); in the system using Mg(OH)_2_, 84% (115,584 L·d^−1^) is treated by SP and 16% (22,016 L·d^−1^) is used to mix the SP treated effluent (BG5) as indicated in Table 5. MgO and Mg(OH)_2_ are chosen in this calculation because of their wide use in environmental applications and the lower NaOH requirement [38] (Table 3).

## 4. Conclusions

The SP treatment using different groups of Mg and P sources recovered nitrogen and improved methane yields, while increasing buffering capacity of acidogenic digester effluents. The treatment using Mg(OH)_2_ and H_3_PO_4_ was the best candidate for TAN removal and methane yield, compared to the control. Residual components after SP, including P and Na, could lead to inhibition of AD and the loss of valuable nutrients. This could be minimized by process control strategies such as addition of extra Mg and lowering the operational pH. SP treatment also contributes to positive economic benefits due to struvite recovery and/or additional methane production.

## Figures and Tables

**Table 1:** Magnesium and phosphorus sources potentially used in struvite precipitation.

	Molar Weight ^1^ (g·mol^−1^)	Solubility ^1^ (g per 100 g Water, 25 °C)	Toxic Residual ^2^	Final Concentration ^3^ (g·L^−1^)	Inhibition Threshold ^4^ (g·L^−1^)
Mg sources					
MgCl_2_	95.2	55.5	Cl	7.1	5.5
MgO	40.3	0.0086 (30 °C)	None	None	None
Mg(OH)_2_	58.3	0.00064	None	None	None
MgCO_3_	84.3	0.0139	None	None	None
MgSO_4_	120.4	37.4	S	3.2	0.05
Mg(NO_3_)_2_	148.3	72.7	N	2.8	1.5
MgHPO_4_	120.3	Slightly soluble	None	None	None
P sources					
H_3_PO_4_	98.0	599.3 (24 °C)	None	None	None
NaH_2_PO_4_	120.0	85.2 (20 °C)	Na	2.3	3.5–5.5
Na_2_HPO_4_	142.0	12.0	Na	4.6	
Na_3_PO_4_	163.9	14.5	Na	6.9	
KH_2_PO_4_	136.1	25.1	K	3.9	2.5–4.5
K_2_HPO_4_	174.2	168.4	K	7.8	
K_3_PO_4_	212.3	105.9	K	11.7	

^1^ Obtained from [12,13]; ^2^ Cl, S, N, Na and K denote the element of Chloride, Sulfur, Nitrogen, Sodium and Potassium, respectively; ^3^ Calculated based on 1.4 g·L^−1^ TAN in the substrate; ^4^ Obtained from [14,15,16].

**Table 2:** Characteristics of effluent from acidogenic digester that is used for struvite precipitation and biochemical methane potential evaluations.

Parameters	Avg. ± Std. Dev.	n ^1^
pH	6.4 ± 0.1	62
TS (g·L^−1^)	2.7 ± 0.2	23
VS (g·L^−1^)	1.7 ± 0.1	23
TSS (g·L^−1^)	1.2 ± 0.2	23
COD (g·L^−1^)	13.3 ± 0.7	25
TAN (g·L^−1^)	1.3 ± 0.1	25
VFA (g acetic acid L^−1^)	10.6 ± 1.8	27

^1^ n = number of data points included; Digester OLR during sampling = 1.2 g COD·L^−1^·d^−1^.

**Table 3:** Total Ammonia Nitrogen (TAN) removal performance of the different magnesium and phosphorus reagent combinations tested.

Group	TAN at T_0_^1^ (g·L^−1^)	TAN at T_20_^1^ (g·L^−1^)			TAN Removal (%)	NaOH Used (g·L^−1^_substrate_)	pH_0_^2^	pH_20_^3^	T_20_^3^ (°C)
		Rep 1	Rep 2	AVG					
G1	1.386	0.345	0.277	0.311	77.5	4.8	5.9	8.5	26.7
G2	1.336	0.714	0.609	0.661	50.5	2.6	6.4	8.5	25.5
G3	1.409	0.159	0.150	0.155	89.0	12.5	3.2	8.4	25.0
G4	1.514	0.177	0.177	0.177	88.3	8.3	5.4	8.7	23.8
G5	1.386	0.377	0.341	0.359	74.1	4.8	5.8	8.5	24.2
G6	1.364	0.632	0.491	0.561	58.8	2.0	6.5	8.9	22.5
G7	1.455	0.364	0.336	0.350	75.9	7.5	5.8	8.5	22.1
G8	1.459	0.318	0.368	0.343	76.5	3.6	6.3	8.4	21.4
G9	1.473	1.386	1.405	1.395	5.2	0.4	6.6	8.2	20.8

^1^ T_0_ and T_20_ denote the reaction time at 0 and 20 min, respectively; ^2^ pH was measured right after adding Mg and P sources; ^3^ These parameters were measured at T_20_.

**Table 4:** Characteristics of untreated (control) and treated substrates by struvite precipitation used for BMP testing.

Parameters ^1^	G0 ^4^	G1	G3	G5	G7	G8
pH	7.1	9.0	8.7	9.2	8.9	8.9
TS (g·L^−1^)	2.9 ± 0.2	14.3 ± 0.1	21.8 ± 0.2	13.9 ± 0.1	16.5 ± 0.2	17.1 ± 0.2
VS (g·L^−1^)	1.8 ± 0.2	5.9 ± 0.2	4.9 ± 0.1	5.9 ± 0.0	5.6 ± 0.1	5.9 ± 0.1
COD (g·L^−1^)	14.7 ± 3.3	13.3 ± 1.2	12.5 ± 2.0	12.2 ± 0.7	11.4 ± 0.1	12.6 ± 1.6
TN (g·L^−1^)	1.5 ± 0.0	0.7 ± 0.0	0.3 ± 0.0	0.5 ± 0.0	0.4 ± 0.0	0.4 ± 0.0
TAN (g·L^−1^)	1.4 ± 0.0	0.6 ± 0.0	0.1 ± 0.0	0.4 ± 0.0	0.3 ± 0.0	0.3 ± 0.0
C/N ^2^	5.0	14.8	22.7	17.0	22.1	21.8
M:A:P ^3^	1:310:8	1:11:11	1:6:6	1:18:18	1:19:19	1:24:23
Micronutrients (ppm or mg·L^−1^)						
Al	1.79	1.53	8.81	<0.50	4.42	0.86
B	<0.20	<0.20	<0.20	<0.20	0.46	0.43
Ca	27.88	33.10	21.22	22.96	37.22	47.74
Cd	<0.10	<0.10	<0.10	<0.10	<0.10	<0.10
Cr	<0.10	<0.10	<0.10	<0.10	<0.10	<0.10
Cu	0.75	1.00	0.41	0.38	0.31	0.27
Fe	15.35	12.39	11.71	10.86	9.83	12.18
K	123.0	121.6	96.2	118.8	124.4	117.5
Mg	7.83	90.44	26.52	42.70	28.60	23.96
Mn	<0.10	<0.10	<0.10	<0.10	<0.10	<0.10
Mo	<0.10	<0.10	<0.10	<0.10	<0.10	<0.10
Na	269	3526	7432	3618	4856	4892
Ni	<0.20	<0.20	<0.20	<0.20	<0.20	<0.20
P	79	1246	195	1005	706	726
Pb	<0.50	<0.50	<0.50	<0.50	<0.50	<0.50
S	54.56	56.94	49.32	52.78	55.84	56.46
Si	7.56	7.60	20.54	10.45	12.64	10.11
Zn	0.40	0.28	<0.10	<0.10	0.19	0.26

^1^ Triplicate for each sample except pH and micronutrients; ^2^ *w*/*w*, dry base; ^3^ Molar ratio of Magnesium to Total ammonia to Phosphorus; ^4^ G0 denotes the untreated substrate.

**Table 5:** Biochemical methane potential (BMP) assay of struvite precipitation (SP) treated substrates.

Treatments ^1^	DI Water (mL)	Inoculum (mL)	Untreated Substrate (mL)	Treated Substrates ^2^ (mL)				
			G0	G1	G3	G5	G7	G8
BI	210	90	0	0	0	0	0	0
BG0	0	90	210	0	0	0	0	0
BG1	0	90	0	210	0	0	0	0
BG3	0	90	81	0	129	0	0	0
BG5	0	90	34	0	0	176	0	0
BG7	0	90	53	0	0	0	157	0
BG8	0	90	51	0	0	0	0	159

^1^ BI is inoculum only control, and BG0 is untreated substrate; BG1, BG3, BG5, BG7 and BG8 denote SP treatment substrates of Group 1, 3, 5, 7 and 8, respectively; ^2^ All the treated substrates were normalized to have 0.6 g·L^−1^ TAN by adding untreated substrate.

**Table 6:** Methane yields and modified Gompertz model parameters of methane production from each treatment.

Parameters	BG0	BG1	BG3	BG5	BG7	BG8
P_m_^1^ (mL)	593.2 ± 213.1	367.9 ± 12.0	516.8 ± 326.5	559.3 ± 22.4	430.7 ± 27.9	400.8 ± 61.3
R_m_^1^ (mL·day^−1^)	2.9 ± 0.4	4.3 ± 0.1	1.6 ± 0.6	2.6 ± 0.4	1.9 ± 0.0	2.8 ± 1.4
λ ^1^ (day)	80.5 ± 9.7	125.7 ± 7.6	20.9 ± 29.6	8.7 ± 4.3	18.3 ± 0.4	68.6 ± 27.5
Methane yield ^2^ (mL·g^−1^ COD_added_)	127.2 ^b^ ± 9.3	116.8 ^b^ ± 4.6	123.3 ^b^ ± 33.7	180.2 ^a^ ± 9.2	125.8 ^b^ ± 2.1	112.6 ^b^ ± 4.8
Methane yield ^2^ (mL·g^−1^ COD_removed_)	263.7 ± 38.1	233.9 ± 7.8	267.5 ± 131.7	277.5 ± 10.2	255.6 ± 7.9	270.6 ± 52.6

^1^ No statistical analysis was done for these parameters because there are only two effective values in BG3, BG5, BG7 and BG8 after the non-linear regression; ^2^ Different letters indicate significant differences (*p* ≤ 0.05).

**Table 7:** Characteristics of the substrate before (B) and after (A) BMP testing.

	COD (g·L^−1^)			TAN (g·L^−1^)			Na (mg·L^−1^)	P (mg·L^−1^)	Cl^−^ (mg·L^−1^)
	B	A	Removal ^1^ (%)	B	A	Removal ^1^ (%)	B	B	A
BG0	10.77	5.45 ± 1.25	49 ± 12	1.42	1.26 ± 0.03	12 ^a^ ± 2	336	80	336
BG1	9.79	4.90 ± 0.25	50 ± 3	0.86	0.79 ± 0.03	7 ^ab^ ± 4	2616	897	335
BG3	9.82	4.69 ± 1.83	52 ± 19	0.85	0.85 ± 0.04	1 ^b^ ± 4	3416	130	3026
BG5	9.30	3.26 ± 0.20	65 ± 2	0.85	0.84 ± 0.03	1 ^b^ ± 3	2300	623	352
BG7	9.04	4.59 ± 0.21	49 ± 2	0.85	0.84 ± 0.01	1 ^b^ ± 1	2736	408	364
BG8	9.66	5.56 ± 0.58	42 ± 6	0.85	0.79 ± 0.03	7 ^ab^ ± 3	2786	422	360

^1^ Different letters indicate significant differences (*p* ≤ 0.05).

**Table 8:** Economic analysis of the struvite precipitation (SP) process.

	Unit Price ($·kg^−1^)	Mass Flow (kg·d^−1^)	Money Flow ($·d^−1^)
Mg source	MgO		
Input			
NaOH	0.44 ^1^	660	287.31
H_3_PO_4_	0.45 ^2^	1348	606.82
MgO	0.55 ^3^	550	302.72
Output			
Struvite (MgNH_4_PO_4_·6H_2_O)	0.84 ^4^	1935	1631.26
Methane ^5^	0.38	0	0
Net benefit ($·d^−1^)			434.42
Mg source	Mg(OH)_2_		
Input			
NaOH	0.44 ^1^	555	241.34
H_3_PO_4_	0.45 ^2^	1133	509.73
Mg(OH)_2_	0.28/0.99 ^3^	670	187.71/663.68
Output			
Struvite (MgNH_4_PO_4_·6H_2_O)	0.84 ^4^	1945	1640.02
Methane ^5^	0.38	97	36.86
Net benefit ($·d^−1^)			738.10/262.13

^1^ Calculated from [36]. ^2^ Calculated from [37]. ^3^ Calculated from [38]. Mg(OH)_2_ is in slurry form ($0.28 kg^−1^) or as powder ($0.99 kg^−1^). ^4^ Average suggested market value of struvite in [39]. ^5^ Calculated from the average residential natural gas price of year 2010–2015 [40]. Unit price and flow rate are in unit of $·m^−3^ and m^3^·d^−1^, respectively. The natural gas containing 100% methane is assumed for easy calculation.

**Table 9:** Dosage of magnesium and phosphorus sources used in experiment 1.

Group	Mg and P Source	Molar Weight (g·mol^−1^)	Amount (g)	Ions Left in the Solutions (mg·L^−1^)	
				Na^+^	Cl^−^
G1	MgO	40.3	1.94	0	0
	H_3_PO_4_ (85%)	98.0	5.6		
G2	MgO	40.3	1.94	2233	0
	NaH_2_PO_4_·H_2_O	138.0	6.7		
G3	MgCl_2_·6H_2_O	203.3	9.86	0	6887
	H_3_PO_4_ (85%)	98.0	5.6		
G4	MgCl_2_·6H_2_O	203.3	9.86	2233	6887
	NaH_2_PO_4_·H_2_O	138.0	6.7		
G5	Mg(OH)_2_	58.3	2.82	0	0
	H_3_PO_4_ (85%)	98.0	5.6		
G6	Mg(OH)_2_	58.3	2.82	2233	0
	NaH_2_PO_4_·H_2_O	138.0	6.7		
G7	MgCO_3_	84.3	4.08	0	0
	H_3_PO_4_ (85%)	98.0	5.6		
G8	MgCO_3_	84.3	4.08	2233	0
	NaH_2_PO_4_·H_2_O	138.0	6.7		
G9	MgHPO_4_·3H_2_O	174.3	8.45	0	0

## Supplementary Materials

Supplementary materials can be accessed at: http://www.mdpi.com/1420-3049/21/8/1011/s1.
