# Supplementary material for: Struvite Precipitation as a Means of Recovering Nutrients and Mitigating Ammonia Toxicity in a Two-Stage Anaerobic Digester Treating Protein-Rich Feedstocks
Source: Molecules. 2016 Aug 3;21(8):1011. doi: 10.3390/molecules21081011 (PMC6273907; doi:10.3390/molecules21081011)
Supplement: Supplementary file 1 [file molecules-21-01011-s001.pdf]

Supplementary Materials: Struvite Precipitation as a Means of Recovering Nutrients and Mitigating Ammonia Toxicity in a Two-Stage Anaerobic Digester Treating Protein-Rich Feedstocks

Shunli Wang, Gary L. Hawkins, Brian H. Kiepper and Keshav C. Das

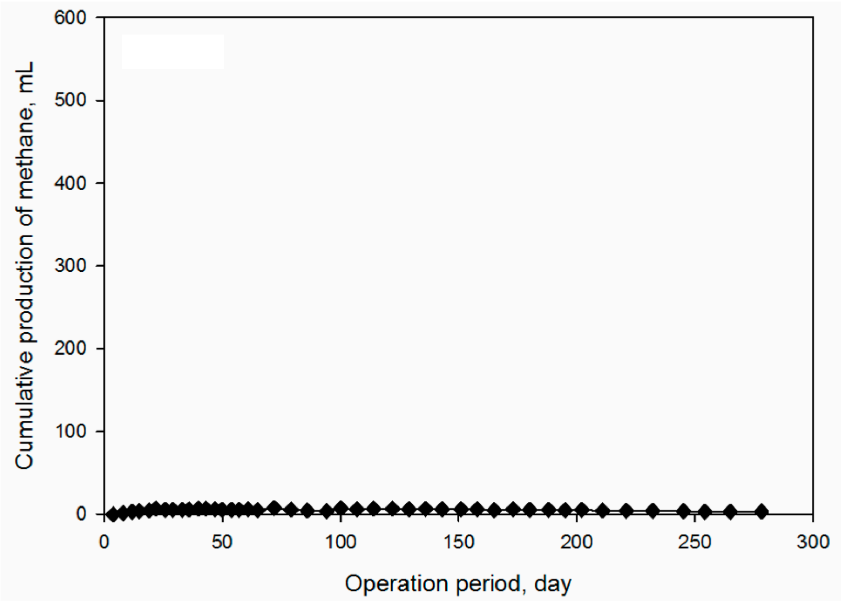

Figure S1. Cumulative methane production of the treatment only containing the inoculum (BI). Each point denotes the average value of three replicates.

Table S1. Preliminary test of acidogenic digester effluent micronutrients.

Parameters	Concentration (ppm or mg·L^{-1})
Aluminum (Al)	13.8
Boron (B)	<0.2
Calcium (Ca)	23.6
Cadmium (Cd)	<0.1
Chromium (Cr)	<0.1
Copper (Cu)	<0.1
Iron (Fe)	28.1
Potassium (K)	160
Magnesium (Mg)	6.04
Manganese (Mn)	<0.1
Molybdenum (Mo)	<0.1
Sodium (Na)	277
Nickel (Ni)	<0.2
Phosphorus (P)	65.5
Lead (Pb)	<0.5
Sulfur (S)	85.1
Silicon (Si)	13.1
Zinc (Zn)	<0.1
